# Impact of degree heterogeneity on the behavior of trapping in Koch networks

**DOI:** 10.1063/1.3493406

**Published:** 2010-11-10

**Authors:** Zhongzhi Zhang, Shuyang Gao, Wenlei Xie

**Affiliations:** School of Computer Science, Fudan University, Shanghai 200433, China and Shanghai Key Lab of Intelligent Information Processing, Fudan University, Shanghai 200433, China

## Abstract

Previous work shows that the mean first-passage time (MFPT) for random walks to a given hub node (node with maximum degree) in uncorrelated random scale-free networks is closely related to the exponent γ of power-law degree distribution $P\left(k\right)∼k^{-\gamma }$, which describes the extent of heterogeneity of scale-free network structure. However, extensive empirical research indicates that real networked systems also display ubiquitous degree correlations. In this paper, we address the trapping issue on the Koch networks, which is a special random walk with one trap fixed at a hub node. The Koch networks are power-law with the characteristic exponent γ in the range between 2 and 3, they are either assortative or disassortative. We calculate exactly the MFPT that is the average of first-passage time from all other nodes to the trap. The obtained explicit solution shows that in large networks the MFPT varies lineally with node number N, which is obviously independent of γ and is sharp contrast to the scaling behavior of MFPT observed for uncorrelated random scale-free networks, where γ influences qualitatively the MFPT of trapping problem.

As a fundamental dynamical process, random walks have received considerable interest from the scientific community. Recent work shows that the key quantity—mean first-passage time (MFPT) for random walks to a given hub node (node with highest degree) on uncorrelated random scale-free networks—is qualitatively reliant on the heterogeneity of network structure. However, in addition to the power-law behavior, most real systems are also characterized by degree correlations. In this paper, we study random walks on a family of recently proposed networks—Koch networks that are transformed from the well-known Koch curves and have simultaneously power-law degree distribution and degree correlations with the power exponent of degree distribution lying between 2 and 3. We explicitly determine the MFPT, i.e., the average of first-passage time to a target hub node averaged over all possible starting positions, and show that the MFPT varies linearly with node number, independent of the inhomogeneity of network structure. Our result indicates that the heterogeneous structure of Koch networks has little impact on the scaling of MFPT in the network family, which is in contrast with the result of MFPT previously reported for uncorrelated stochastic scale-free graphs.

## I. INTRODUCTION

In the past decade, a lot of endeavors have been devoted to characterize the structure of real systems from the view point of complex networks,^1–4^ where nodes represent system elements and edge interactions or relations between them. One of the most important findings of extensive empirical studies is that a wide variety of real networked systems exhibits scale-free behavior,^5^ characterized by a power-law degree distribution $P\left(k\right)∼k^{-\gamma }$ with degree exponent γ lying in the interval of [2,3]. Networks with such broad tail distribution are called scale-free networks, which display inhomogeneous structure encoded in the exponent γ: the less the exponent γ, the stronger the inhomogeneity of the network structure, and vice versa. The heterogeneous structure critically influences many other topological properties. For instance, it has been shown that in uncorrelated random scale-free networks with node number N (often called network order), their average path length (APL) (Ref. 6) relies on γ:^7,8^ for γ=3, d(N)∼ln N, while for 2≤γ<3, d(N)∼ln ln N.

The power-law degree distribution also radically affects the dynamical processes running on scale-free networks,^9^ such as disease spreading,^10^ percolation,^11^ and so on. Among various dynamics, random walks are an important one that have a wide range of applications^12–14^ and have received considerable attention.^15–18^ Recently, MFPT for random walks to a given target point in graphs, averaged over all source points, has been extensively studied.^19–23^ A striking finding is that MFPT to a hub node (node with highest degree) in scale-free networks scales sublinearly with network order,^24–27^ the root of which is assumed to be the structure heterogeneity of the networks. In particularly, it has been reported^28^ that in uncorrelated random scale-free networks, the MFPT F(N) scales with network order N as $F\left(N\right)∼N^{\left(\gamma -2)/(\gamma -1\right)}$. However, real networks exhibit ubiquitous degree correlations among nodes, they are either assortative or disassortative.^29^ Then, an interesting question arises naturally: whether the relation governing MFPT and degree exponent γ in uncorrelated scale-free networks is also valid for their correlated counterparts.

In this paper, we study analytically random walks in the Koch networks^30,31^ that are controlled by a positive-integer parameter m. This family of networks is scale-free with the degree exponent γ lying between 2 and 3, and it may be either disassortative (m>1) or uncorrelated (m=1). We focus on the trapping problem, a particular case of random walks with a fixed trap located at a hub node. We derive exactly the MFPT that is the average of first-passage time (FPT) from all starting nodes to the trap. The obtained explicit formula displays that in large networks with N nodes, the MFPT grows linearly with N, which is independent of γ and, showing that the structure inhomogeneity has no quantitative influence on the MFPT to the hub in Koch networks, which lies in their symmetric structure and other special features and is quite different from the result previously reported for uncorrelated random scale-free networks. Our work deepens the understanding of random walks occurring on scale-free networks.

## II. CONSTRUCTION AND PROPERTIES OF KOCH NETWORKS

**FIG. 1.**
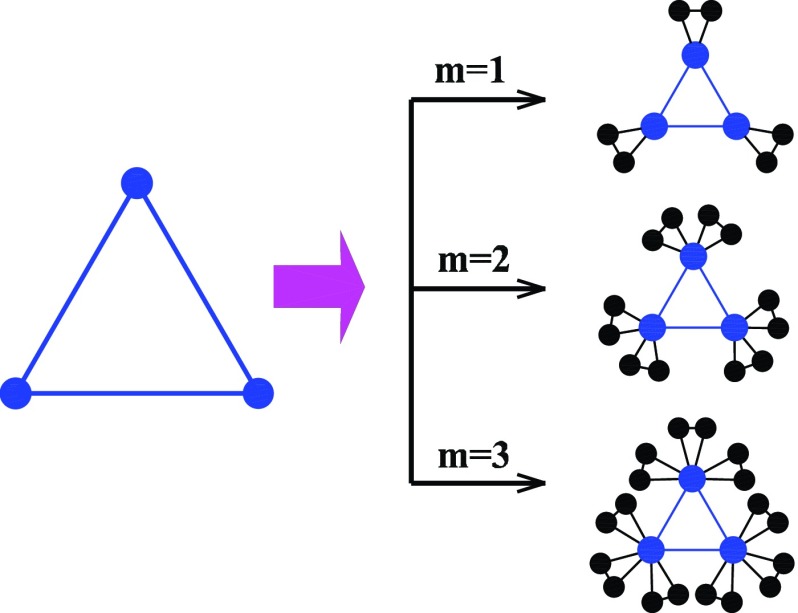
Iterative construction method for the Koch networks.

**FIG. 2.**
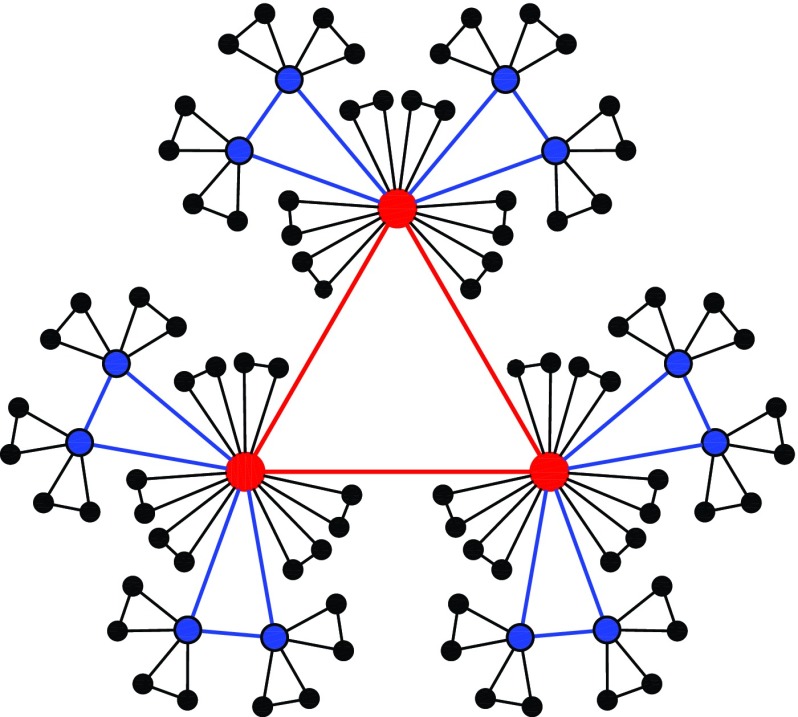
A network corresponding to the case of m=2.

The Koch networks governed by a parameter m are derived from the famous Koch curves,^32,33^ which are constructed in an iterative way.^30,31^ Let $K_{m,t}$ denote the Koch networks after t iterations. Then, the networks can be generated as follows: initially (t=0), $K_{m,0}$ is a triangle. For t≥1, $K_{m,t}$ is obtained from $K_{m,t-1}$ by adding m groups of nodes for each of the three nodes of every existing triangles in $K_{m,t-1}$. Each node group consists of two nodes, both of which and their “father” node are connected to one another shaping a new triangle. That is to say, to get $K_{m,t}$ from $K_{m,t-1}$, one can replace each existing triangle in $K_{m,t-1}$ by the connected clusters on the right-hand side of Fig. 1. Figure 2 shows a network corresponding to m=2 after several iterations.

By construction, the total number of triangles $L_{△}\left(t\right)$ at iteration t is $L_{△}\left(t\right)={\left(3m+1\right)}^{t}$, and the number of nodes created at iteration t is $L_{v}\left(t\right)=6mL_{△}\left(t-1\right)=6m{\left(3m+1\right)}^{t-1}$. Then, the total number of nodes $N_{t}$ present at step t is

$$N_{t}=\sum_{t_{i}=0}^{t}L_{v}\left(t_{i}\right)=2{\left(3m+1\right)}^{t}+1.$$ (1)

Let $k_{i}\left(t\right)$ be the degree of a node i at time t, which is added to the networks at iteration (step) $t_{i}\left(t_{i}\geq 0\right)$. Then, $k_{i}\left(t_{i}\right)=2$. Let $L_{△}\left(i,t\right)$ denote the number of triangles involving node i at step t. According to the construction algorithm, each triangle involving node i at a given step will give birth to m new triangles passing by node i at next step. Thus, $L_{△}\left(i,t\right)=\left(m+1\right)L_{△}\left(i,t-1\right)={\left(m+1\right)}^{t-t_{i}}$. Moreover, it is easy to have $k_{i}\left(t\right)=2L_{△}\left(i,t\right)$, i.e.,

$$k_{i}\left(t\right)=2L_{△}\left(i,t\right)=2{\left(m+1\right)}^{t-t_{i}},$$ (2)

which implies

$$k_{i}\left(t\right)=\left(m+1\right)k_{i}\left(t-1\right).$$ (3)

The Koch networks present some common features of real systems.^1,2^ They are scale-free, having a power-law degree distribution $P\left(k\right)∼k^{-\gamma }$ with γ=1+ln(3m+1)/ln(m+1) belonging to the range between 2 and 3. Thus, parameter m controls the extent of heterogeneous structure of Koch networks with larger m corresponding to more heterogeneous structure. They have small-world effect with a low APL and a high clustering coefficient. In addition, their degree correlations can be also determined. For m=1, they are completely uncorrelated, while for other values of m, the Koch networks are disassortative.

## III. RANDOM WALKS WITH A TRAP FIXED ON A HUB NODE

**FIG. 3.**
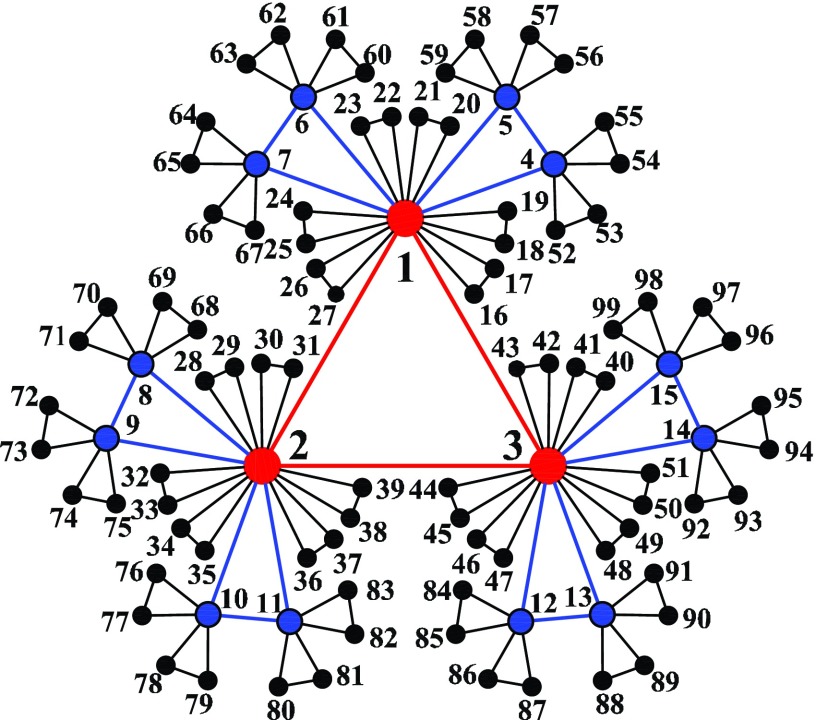
Labels of all nodes in $K_{2,2}$.

After introducing the construction and structural properties of the Koch networks, we continue to investigate random walks^34^ performing on them. Our aim is to uncover how topological features, especially degree correlations, influence the behavior of a simple random walk on Koch networks with a single trap or a perfect absorber stationed at a given node with highest degree. At each step, the walker located on a given node moves uniformly to any of its nearest neighbors. To facilitate the description, we label all the nodes in $K_{m,t}$ as follows. The initial three nodes in $K_{m,0}$ have labels 1, 2, and 3, respectively. In each new generation, only the newly created nodes are labeled, while all old nodes hold the labels unchanged. That is to say, the new nodes are labeled consecutively as M+1,M+2,…,M+ΔM, with M being the number of all pre-existing nodes and ΔM the number of newly created nodes. Eventually, every node has a unique labeling: at time t all nodes are labeled continuously from 1 to $N_{t}=2{\left(3m+1\right)}^{t}+1$, see Fig. 3. We locate the trap at node 1, denoted by $i_{T}$.

We will show that the particular selection of the trap location makes it possible to compute analytically the relevant quantity of the trapping process, i.e., mean first-passage time. Let $F_{i}^{\left(t\right)}$ denote the first-passage time of node i in $K_{m,t}$ except the trap $i_{T}$, which is the expected time for a walker starting from i to visit the trap for the first time. The mean of FPT $F_{i}^{\left(t\right)}$ over all nontrap nodes in $K_{m,t}$ is MFPT, denoted by ${\left\langle F\right\rangle }_{t}$, the determination of which is a main object of the section. To this end, we first establish the scaling relation governing the evolution of $F_{i}^{\left(t\right)}$ with generation t.

### A. Evolution scaling for first-passage time

We begin by recording the numerical values of $F_{i}^{\left(t\right)}$ for the case of m=2. Clearly, for all t≥0, $F_{1}^{\left(0\right)}=0$; for t=0, it is trivial, and we have $F_{2}^{\left(0\right)}=F_{3}^{\left(0\right)}=2$. For t≥1, the values of $F_{i}^{\left(t\right)}$ can be obtained numerically but exactly via computing the inversion of a matrix, which will be discussed in the following text. Here we only give the values of computation. In the generation n=1, by symmetry we have $F_{2}^{\left(1\right)}=F_{3}^{\left(1\right)}=14$, $F_{4}^{\left(1\right)}=F_{5}^{\left(1\right)}=F_{6}^{\left(1\right)}=F_{7}^{\left(1\right)}=2$, and $F_{8}^{\left(1\right)}=F_{9}^{\left(1\right)}=\cdots =F_{15}^{\left(1\right)}=16$. Analogously, for t=2, the numerical solutions are $F_{2}^{\left(2\right)}=F_{3}^{\left(2\right)}=98$, $F_{4}^{\left(2\right)}=F_{5}^{\left(2\right)}=F_{6}^{\left(2\right)}=F_{7}^{\left(2\right)}=14$, $F_{8}^{\left(2\right)}=F_{9}^{\left(2\right)}=\cdots =F_{15}^{\left(2\right)}=112$, $F_{16}^{\left(2\right)}=F_{17}^{\left(2\right)}=\cdots =F_{27}^{\left(2\right)}=2$, $F_{28}^{\left(2\right)}=F_{29}^{\left(2\right)}=\cdots =F_{51}^{\left(2\right)}=100$, $F_{52}^{\left(2\right)}=F_{53}^{\left(2\right)}=\cdots =F_{67}^{\left(2\right)}=16$, and $F_{68}^{\left(2\right)}=F_{69}^{\left(2\right)}=\cdots =F_{99}^{\left(2\right)}=114$. Table I lists the numerical values of $F_{i}^{\left(t\right)}$ for some nodes up to t=5.

The numerical values reported in Table I show that for any node i, its FPT satisfies the relation $F_{i}^{\left(t+1\right)}=\left(3m+1\right)F_{i}^{\left(t\right)}$. In other words, upon growth of Koch networks from generation t to t+1, the FPT of any node increases to 3m+1 times. For example, $F_{2}^{\left(5\right)}=7F_{2}^{\left(4\right)}=7^{2}F_{2}^{\left(3\right)}=7^{3}F_{2}^{\left(2\right)}=7^{4}F_{2}^{\left(1\right)}=7^{5}F_{2}^{\left(0\right)}=33\;614$, $F_{8}^{\left(5\right)}=7F_{8}^{\left(4\right)}=7^{2}F_{8}^{\left(3\right)}=7^{3}F_{8}^{\left(2\right)}=7^{4}F_{8}^{\left(1\right)}=38\;416$, and so forth. This scaling is a basic property of random walks on the family of Koch networks, which can be established based on the following arguments.

**FIG. 4.**
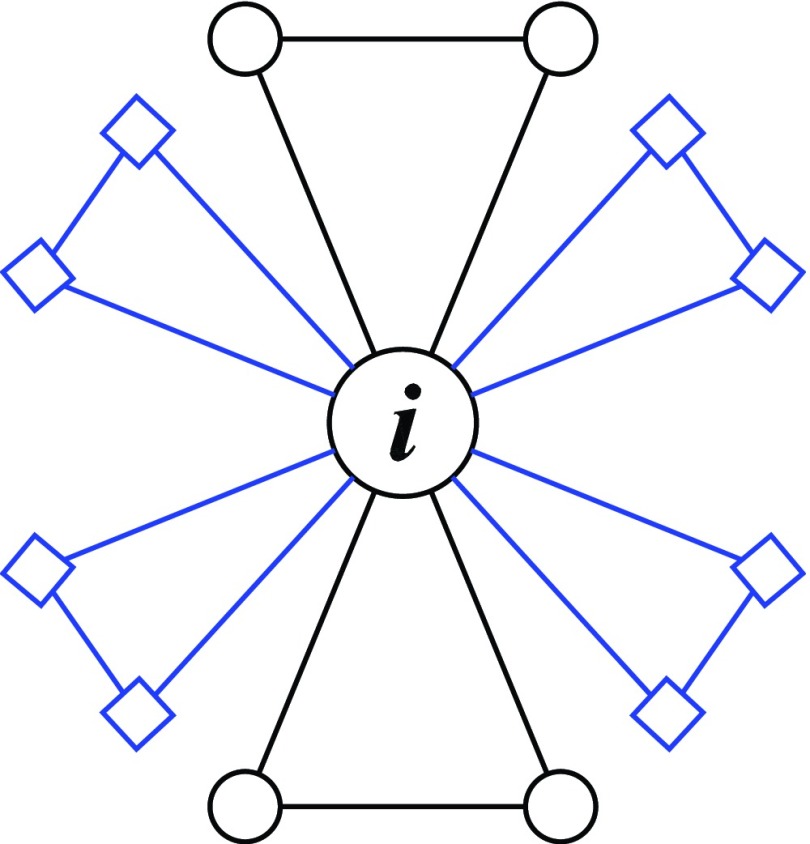
Growth of first-passage time in going from $K_{m,t}$ to $K_{m,t+1}$ in the case of m=2. Node $i∊K_{m,t}$ has $k_{i}$ neighbor nodes in generation t (○) and $mk_{i}$ new neighbor nodes in generation t+1 (◻). A new neighbor of node i has a degree of 2, and is simultaneously linked to another new neighbor of i.

Examine an arbitrary node i in the Koch networks $K_{m,t}$. Equation (3) shows that upon growth of the networks from generation t to t+1, the degree $k_{i}$ of node i grows by m times, i.e., it increases from $k_{i}$ to $\left(m+1\right)k_{i}$. Let A denote the FPT for going from node i to any of its $k_{i}$ old neighbors, and let B be FPT for starting from any of the $mk_{i}$ new neighbors of node i to one of its $k_{i}$ old neighboring nodes. Then the following equations can be established (see Fig. 4):

{A=1m+1+mm+1(1+B),B=12(1+A)+12(1+B),} (4)

which yield A=3m+1. This indicates that when the networks grow from generation t to t+1, the FPT from any node $i\left(i∊K_{m,t}\right)$ to any node $j\left(j∊K_{m,t+1}\right)$ increases on average 3m times. Then, we have $F_{i}^{\left(t+1\right)}=\left(3m+1\right)F_{i}^{\left(t\right)}$. For explanation, see Refs. 35 and 36 and related references therein. The obtained relation for FPT is very useful for the following derivation of MFPT.

### B. Explicit expression for mean first-passage time

Having obtained the scaling dominating the evolution for FPT, we now draw upon this relation to determine the MFPT, with an aim to derive an explicit solution. For the sake of convenient description of computation, we represent the set of nodes in $K_{m,t}$ as $\Theta_{t}$, and denote the set of nodes created at generation t by ${\bar{\Theta }}_{t}$. Evidently, the relation $\Theta_{t}={\bar{\Theta }}_{t}\cup \Theta_{t-1}$ holds. In addition, for any r≤t, we define the two following variables:

$$F_{r,\text{tot}}^{\left(t\right)}=\sum_{i∊\Theta_{r}}F_{i}^{\left(t\right)}$$ (5)

and

$${\bar{F}}_{r,\text{tot}}^{\left(t\right)}=\sum_{i∊{\bar{\Theta }}_{r}}F_{i}^{\left(t\right)}.$$ (6)

Then, we have

$$F_{t,\text{tot}}^{\left(t\right)}=F_{t-1,\text{tot}}^{\left(t\right)}+{\bar{F}}_{t,\text{tot}}^{\left(t\right)}=\left(3m+1\right)F_{t-1,\text{tot}}^{\left(t-1\right)}+{\bar{F}}_{t,\text{tot}}^{\left(t\right)}$$ (7)

and

$${\left\langle F\right\rangle }_{t}=\frac{F_{t,\text{tot}}^{\left(t\right)}}{N_{t}-1}.$$ (8)

Thus, to explicitly determine the quantity ${\left\langle F\right\rangle }_{t}$, one should first find $F_{t,\text{tot}}^{\left(t\right)}$, which can be reduced to determining ${\bar{F}}_{t,\text{tot}}^{\left(t\right)}$. Next, we will show how to solve the quantity ${\bar{F}}_{t,\text{tot}}^{\left(t\right)}$.

**FIG. 5.**
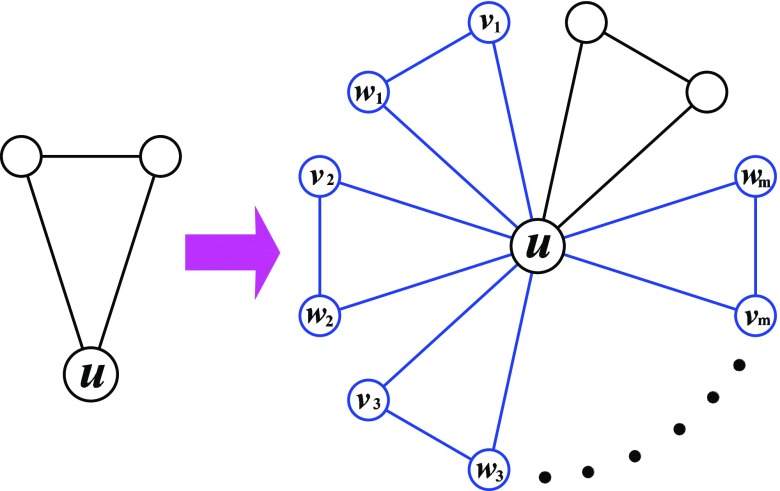
Illustration showing the relation of the first-passage times for each pair of two new nodes ($v_{x}$ and $w_{x}$ with x=1,2,…, or m) and the old node u as one point of the triangle generating the new nodes.

By construction, at a given generation, for each triangle passing by node u, it will generate m new triangles involving u (see Fig. 5). For each of the m new triangles, the first-passage times for its two new nodes ($v_{x}$ and $w_{x}$) and that of its old node u follow the relations,

{F(vx)=1+12[F(wx)+F(u)],F(wx)=1+12[F(vx)+F(u)].} (9)

In Eq. (9), F(s) represents the expected time of a particle to first visit the trap node, given that it starts from node s. Equation (9) yields

$$F\left(v_{x}\right)+F\left(w_{x}\right)=4+2F\left(u\right).$$ (10)

Summing Eq. (10) over all the $L_{△}\left(t\right)={\left(3m+1\right)}^{t}$ old triangles pre-existing at the generation t and the three old nodes of each of the $L_{△}\left(t\right)$ triangles, we obtain

$${\bar{F}}_{t+1,\text{tot}}^{\left(t+1\right)}=3\cdot 4\cdot mL_{△}\left(t\right)+\sum_{i∊\Theta_{t}}\left(2mL_{△}\left(i,t\right)\cdot F_{i}^{\left(t+1\right)}\right)=12m{\left(3m+1\right)}^{t}+2m{\bar{F}}_{t,\text{tot}}^{\left(t+1\right)}+2m\left(m+1\right){\bar{F}}_{t-1,\text{tot}}^{\left(t+1\right)}+\cdots +2m{\left(m+1\right)}^{t-1}{\bar{F}}_{1,\text{tot}}^{\left(t+1\right)}+2m{\left(m+1\right)}^{t}{\bar{F}}_{0,\text{tot}}^{\left(t+1\right)}.$$ (11)

For instance, in $K_{2,2}$ (see Fig. 3), ${\bar{F}}_{2,\text{tot}}^{\left(2\right)}$ can be expressed as

$${\bar{F}}_{2,\text{tot}}^{\left(2\right)}=\sum_{i=16}^{99}F_{i}^{\left(2\right)}=1176+12{\bar{F}}_{1,\text{tot}}^{\left(2\right)}+36{\bar{F}}_{0,\text{tot}}^{\left(2\right)}.$$ (12)

Now, we can determine ${\bar{F}}_{t,\text{tot}}^{\left(t\right)}$ through a recurrence relation, which can be obtained easily. From Eq. (12), it is not difficult to write out ${\bar{F}}_{t+2,\text{tot}}^{\left(t+2\right)}$ as

$${\bar{F}}_{t+2,\text{tot}}^{\left(t+2\right)}=12m{\left(3m+1\right)}^{t+1}+2m{\bar{F}}_{t+1,\text{tot}}^{\left(t+2\right)}+2m\left(m+1\right){\bar{F}}_{t,\text{tot}}^{\left(t+2\right)}+\cdots +2m{\left(m+1\right)}^{t}{\bar{F}}_{1,\text{tot}}^{\left(t+2\right)}+2m{\left(m+1\right)}^{t+1}{\bar{F}}_{0,\text{tot}}^{\left(t+2\right)}.$$ (13)

Equation (13) minus Eq. (11) times (m+1)(3m+1) and using the relation $F_{i}^{\left(t+2\right)}=\left(3m+1\right)F_{i}^{\left(t+1\right)}$, we have

$${\bar{F}}_{t+2,\text{tot}}^{\left(t+2\right)}={\left(3m+1\right)}^{2}{\bar{F}}_{t+1,\text{tot}}^{\left(t+1\right)}-12m^{2}{\left(3m+1\right)}^{t+1}.$$ (14)

Making use of the initial condition ${\bar{F}}_{1,\text{tot}}^{\left(1\right)}=24m^{2}+20m$, Eq. (14) is solved inductively to yield

$${\bar{F}}_{t,\text{tot}}^{\left(t\right)}=4m{\left(3m+1\right)}^{t-1}+\left(24m^{2}+16m\right){\left(3m+1\right)}^{2t-2}.$$ (15)

Inserting Eq. (15) for ${\bar{F}}_{t,\text{tot}}^{\left(t\right)}$ into Eq. (8), we have

$$F_{t,\text{tot}}^{\left(t\right)}=\left(3m+1\right)F_{t-1,\text{tot}}^{\left(t-1\right)}+4m{\left(3m+1\right)}^{t-1}+\left(24m^{2}+16m\right){\left(3m+1\right)}^{2t-2}.$$ (16)

Since $F_{0,\text{tot}}^{\left(0\right)}=4$, we can resolve Eq. (16) by induction to obtain

$$F_{t,\text{tot}}^{\left(t\right)}=\frac{4}{3}{\left(3m+1\right)}^{t-1}\left[\left(6m+4\right){\left(3m+1\right)}^{t}+3mt+3m-1\right].$$ (17)

By plugging Eq. (17) into Eq. (8), we obtain the closed-form solution to the MFPT for random walks on the Koch networks with an immobile trap stationed at a hub node,

$${\left\langle F\right\rangle }_{t}=\frac{2}{3\left(3m+1\right)}\left[\left(6m+4\right){\left(3m+1\right)}^{t}+3mt+3m-1\right].$$ (18)

### C. Numerical calculations

We have corroborated our analytical formula for MFPT provided by Eq. (18) against direct numerical calculations via inverting a matrix.^37^ Indeed, the Koch network family $K_{m,t}$ can be represented by its adjacency matrix $A_{t}$ of an order $N_{t}\times N_{t}$, the element $a_{ij}\left(t\right)$ of which is either 1 or 0 defined as follows: $a_{ij}\left(t\right)=1$ if nodes i and j are directly connected by a link, and $a_{ij}\left(t\right)=0$ otherwise. Then the degree $d_{i}\left(t\right)$ of node i in $K_{m,t}$ is given by $d_{i}\left(t\right)=\sum_{j}^{N_{t}}a_{ij}\left(t\right)$, the diagonal degree matrix $Z_{t}$ associated with $K_{m,t}$ is $Z_{t}=\text{diag}\left(d_{1}\left(t\right),d_{2}\left(t\right),\ldots ,d_{i}\left(t\right),\ldots ,d_{N_{t}}\left(t\right)\right)$, and the normalized Laplacian matrix of $K_{m,t}$ is provided by $L_{t}=I_{t}-Z_{t}^{-1}A_{t}$, in which $I_{t}$ is the $N_{t}\times N_{t}$ identity matrix.

Note that the random walks considered above are in fact a Markovian process, and the fundamental matrix of Markov chain representing such unbiased random walks is the inverse of a submatrix of $L_{t}$, denoted by ${\bar{L}}_{t}$ that is obtained by removing the first row and column of $L_{t}$ corresponding to the trap node. According to previous result,^37^ the FPT $F_{i}^{\left(t\right)}$ can be expressed in terms of the entry ${\bar{l}}_{ij}^{-1}\left(t\right)$ of ${\bar{L}}_{t}^{-1}$ as

$$F_{i}^{\left(t\right)}=\sum_{j=2}^{N_{t}}{\bar{l}}_{ij}^{-1}\left(t\right),$$ (19)

where ${\bar{l}}_{ij}^{-1}\left(t\right)$ is the expected times that the walk visit node j, given that it starts from node i.^37^ Using Eq. (19) we can determine $F_{i}^{\left(t\right)}$ numerically but exactly for different nontrap nodes at various generation t, as listed in Table I.

By definition, the MFPT ${\left\langle F\right\rangle }_{t}$ that is the mean of $F_{i}^{\left(t\right)}$ over all initial nontrap nodes in $K_{m,t}$ reads as

$${\left\langle F\right\rangle }_{t}=\frac{1}{N_{t}-1}\sum_{i=2}^{N_{t}}F_{i}^{\left(t\right)}=\frac{1}{2{\left(3m+1\right)}^{t}}\sum_{i=2}^{N_{t}}\sum_{j=2}^{N_{t}}{\bar{l}}_{ij}^{-1}\left(t\right).$$ (20)

**FIG. 6.**
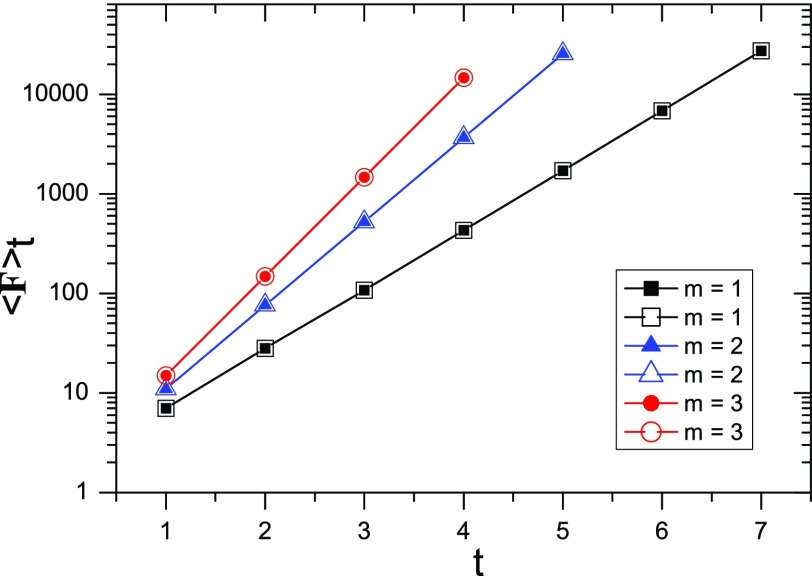
Mean first-passage time ${\left\langle F\right\rangle }_{t}$ as a function of the generation t on a semilogarithmic scale for different values of m. The empty symbols represent the numerical results obtained by direct calculation from Eq. (20), while the filled symbols correspond to the rigorous values provided by Eq. (18).

In Fig. 6, we compare the analytical results given by Eq. (18) and the numerical results obtained by Eq. (20) for various t and m. Figure 6 shows that the analytical and numerical values for ${\left\langle F\right\rangle }_{t}$ are in full agreement with each other. This agreement serves as a test of our analytical formula.

### D. Dependence of mean first-passage time on network order

Below we will show how to express ${\left\langle F\right\rangle }_{t}$ as a function of network order $N_{t}$, with the aim of obtaining the relation between these two quantities. Recalling Eq. (1), we have ${\left(3m+1\right)}^{t}=(N_{t}-1)/2$ and $t=[\text{ln}\left(N_{t}-1\right)-\text{ln}\;2]/\text{ln}\left(3m+1\right)$. Thus, Eq. (18) can be recast in terms of $N_{t}$ as

$${\left\langle F\right\rangle }_{t}=\frac{2\left(3m+2\right)}{3\left(3m+1\right)}\left(N_{t}-1\right)+\frac{2m\left[\text{ln}\left(N_{t}-1\right)-\text{ln}\;2\right]}{\left(3m+1\right)\text{ln}\left(3m+1\right)}\frac{2\left(3m-1\right)}{3\left(3m+1\right)}.$$ (21)

In the thermodynamic limit $\left(N_{t}\to \infty \right)$, we have

$${\left\langle F\right\rangle }_{t}\approx \frac{2\left(3m+2\right)}{3\left(3m+1\right)}\left(N_{t}-1\right)∼N_{t},$$ (22)

showing that the MFPT grows linearly with increasing order of the Koch networks. Equations (21) and (22) imply that although for different m the MFPT of whole family of Koch networks is quantitatively different, it exhibits the same scaling behavior despite the distinct extent of structure inhomogeneity of the networks, which may be attributed to the symmetry and particular properties of the networks studied.

It is known that the exponent γ characterizing the inhomogeneity of networks affects qualitatively the scaling of MFPT for diffusion in random uncorrelated scale-free networks.^28^ Concretely, in random uncorrelated scale-free networks with large order N, the MFPT F(N) grows sublinearly or linearly with network order as $F\left(N\right)∼N^{\left(\gamma -2)/(\gamma -1\right)}$ for all γ>2, which strongly depends on γ. However, as shown above, in the whole family of Koch networks, the MFPT displays a linear dependence on network order, which is independent of γ, showing that the inhomogeneity of structure has no quantitative impact on the scaling behavior of MFPT for trapping process in Koch networks. Our obtained result means that the scaling observed for MFPT in the literature^28^ is not a generic feature of all scale-free networks, at least it is not valid for the Koch networks, even for the case of m=1 when network is uncorrelated.

## IV. CONCLUSIONS

Power-law degree distribution and degree correlations play a significant role in the collective dynamical behaviors on scale-free networks. In this paper, we have investigated the trapping issue, concentrating on a particular case with the trap fixed on a node with highest degree on the Koch networks that display synchronously a heavy-tailed degree distribution with general exponent γ∊[2,3] and degree correlations. We obtained explicitly the formula for MFPT to the trapping node, which scales lineally with network order, independent of the exponent γ. Our result shows that structural inhomogeneity of the Koch networks has no essential effect on the scaling of MFPT for the trapping issue, which departs a little from that one expects and is as compared with the scaling behavior reported for stochastic uncorrelated scale-free networks. Thus, caution must be taken when making a general statement about the dependence of MFPT for trapping issue on the inhomogeneous structure of scale-free networks. Finally, it should be also mentioned that both random uncorrelated networks and the Koch networks addressed here cannot well describe real systems, future work should focus on trapping problem on those networks better mimicking realties. Anyway, our work provides some insight to better understand the trapping process in scale-free graphs.

## Figures and Tables

**Table I.** First-passage time $F_{i}^{\left(t\right)}$ for a random walker starting from node i in $K_{m,t}$ for different t. Note that, thanks to the symmetry, nodes in the same column are equivalent to one another, since they have the same FPT.

t/i	2–3	4–7	8–15	16–27	28–51	52–67	68–99
0	2						
1	14	2	16				
2	98	14	112	2	100	16	114
3	686	98	784	14	700	112	798
4	4802	686	5488	98	4900	784	5586
5	33 614	4602	38 416	686	34 300	5488	39 102
